# Sorption, separation and recycling of ammonium in agricultural soils: A viable application for magnetic biochar?

**DOI:** 10.1016/j.scitotenv.2021.151440

**Published:** 2022-03-15

**Authors:** Max D. Gillingham, Rachel L. Gomes, Rebecca Ferrari, Helen M. West

**Affiliations:** a Faculty of Engineering, University of Nottingham, Nottingham, United Kingdom; b School of Biosciences, University of Nottingham, Nottingham, United Kingdom

**Keywords:** Magnetic biochar, Circular economy, Pollution, Remediation, Nitrogen sorption, Recycling

## Abstract

Recent research on the magnetisation of biochar, a carbon-based material that can be used as a sorbent, has opened novel opportunities in the field of environmental remediation, as incorporating magnetic particles into biochar can simplify subsequent separation. This could offer a sustainable circular economy-based solution in two areas of waste management; firstly, pyrolysis of agricultural waste for magnetic biochar synthesis could reduce greenhouse gas emissions derived from traditional agricultural waste processing, such as landfill and incineration, while secondly, application of magnetic biochar to remove excess nitrogen from soils (made possible through magnetic separation) could provide opportunities for this pollutant to be used as a recycled fertiliser. While sorption of pollutants by magnetic biochar has been researched in wastewater, few studies have investigated magnetic biochar use in polluted soils. Nitrogen pollution (e.g. NH_4_^+^), stemming from agricultural fertiliser management, is a major environmental and economic issue that could be significantly reduced before losses from soils occur. This review demonstrates that the use of magnetic biochar tailored to NH_4_^+^ adsorption has potential to remove (and recycle for reuse) excess nitrogen from soils. Analysis of research into recovery of NH_4_^+^ by sorption/desorption, biochar magnetisation and biochar-soil interactions, suggests that this is a promising application, but a more cohesive, interdisciplinary approach is called for to elucidate its feasibility. Furthermore, research shows variable impacts of biochar upon soil chemistry and biology, such as pH and microbial diversity. Considering wide concerns surrounding global biodiversity depletion, a more comprehensive understanding of biochar-soil dynamics is required to protect and support soil ecosystems. Finally, addressing research gaps, such as optimisation and scaling-up of magnetic biochar synthesis, would benefit from systems thinking approaches, ensuring the many complex considerations across science, industry, policy and economics are connected by circular-economy principles.

## Highlights

• The complex issue of nitrogen pollution requires novel solutions.
• Magnetic biochar (MB) may effectively sorb ammonium (NH_4_^+^).
• The plausibility of a novel MB application is discussed: recycling NH_4_^+^ from soils.
• It is concluded that the strategy is viable, dependent on magnetic separation.
• Future research must be interdisciplinary and inclusive of agro-ecology.

## Graphical abstract

**Figure:**
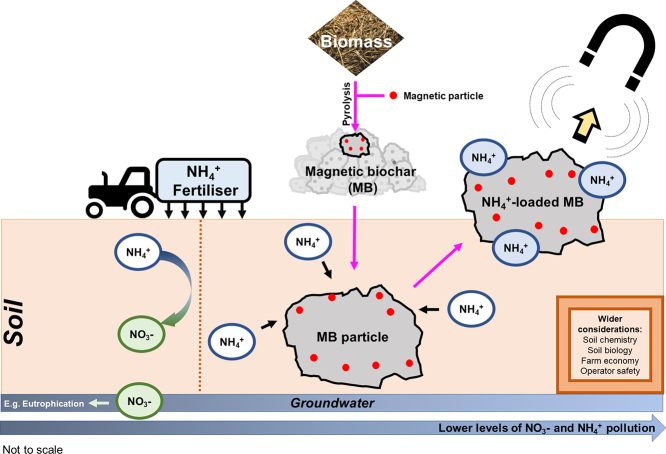

## 1 Introduction

In recent decades, soil health has come to the forefront of global sustainability and food security concerns, with degradation and pollution of agricultural soils deemed to be as serious as the climate crisis and biodiversity loss (FAO and UNEP, 2021). Soil pollution has been ranked third in importance for threats to soil health in Europe and Eurasia (FAO, 2015), and in England and Wales, degraded soils are estimated to cost approximately £1.2bn per year (Graves et al., 2015), with pollution highlighted as being one of the major negative causes (Environment Agency, 2019). In addition, there are countless wider environmental issues caused directly and indirectly by soil pollution in the UK, such as ecotoxicity, eutrophication, and greenhouse gas emissions (Air Quality Expert Group, 2018). The challenge to reduce soil pollution therefore demands development and improvement of evidence-based soil management strategies.

Pollution originating from fertiliser use is a prevalent global issue, and despite small successes in reduction (mainly through legislation), an alarming level of pollution still exists in the UK (Hall et al., 2018). Although not the sole cause (urbanisation is another important factor, for example), agriculture is often the source of diffuse nitrogen (N) pollution and strategies should aim to prevent loss of N from farms. Fertiliser overuse can lead to an accumulation of reactive nitrogen forms in soils (ammonium (NH_4_^+^), nitrite (NO_2_^−^) and nitrate (NO_3_^−^)) (Erisman et al., 2011). These compounds can be leached or run-off from soils and pollute surface and ground-waters and are a major cause of eutrophication observed in surface waters, with over 50% of nitrogen in UK surface water being agriculturally sourced (Hughes et al., 2008). In addition, denitrification can lead to the release of gasses including nitrous oxide (N_2_O), a major greenhouse gas (GHG) (Richardson et al., 2009). As UK policy aims for net zero GHG emissions by 2050, the agricultural sector, which is responsible for approximately 10% of GHG emissions (DBEIS, 2020), has been targeted as a key industry where emissions must be reduced (CCC, 2020). Reducing the levels of surplus nitrogen in soils should therefore be a focus of remediation – a desirable outcome would be maximising the efficiency of a farm's nitrogen use, by capturing nitrogen from heavily polluted soils, or soils where pollution of water is particularly likely, and recycling it to deficient soils. As a result, the agricultural sector could work towards net zero nitrogen emissions, a key step in resolving the GHG issue (Butterbach-Bahl et al., 2011).

Loss of nitrogen from soils manifests as a major financial impact for farmers - the average surplus of nitrogen on agricultural land in 2019 was estimated as 76 kg hectare^−1^ (DEFRA, 2020a), a large proportion of which is expected to leach from the soil. Since the average price for UK-produced ammonium nitrate fertiliser in September 2020 was £257 t^−1^ (AHDB, 2020), a 76 kg ha^−1^ surplus would equate to £20 ha^−1^. On the average English farm size of 81 ha (DEFRA, 2020b), this would cost the farmer £1620 per year. Despite this, the full issue is often underappreciated. For example, Carswell et al. (2019) showed that although urea is a cheaper fertiliser compared to urease inhibited urea or ammonium nitrate, its cost per kg is higher when N losses via NH_3_ and N_2_O are taken into account. This is increased even further when societal costs (for example, damage to ecosystems) are incorporated. Nitrogen use in agriculture therefore needs to be approached in a more dynamic manner, and its recapture and recycling could be a key strategy in reducing its financial and environmental damage.

Current remediation strategies for agriculturally derived nitrogen include reducing the nitrogen load in already-contaminated water bodies or, occasionally, through novel methods to remediate eutrophic water (e.g. phytoremediation (Chen et al., 2017)). However, preventing nitrogen from escaping from agricultural soils is a more cost-effective strategy – a case study from Germany found preventative measures (such as more organic farming, fewer mineral fertilisers, and using buffer strips) were five times cheaper than denitrification to attain acceptable nitrate groundwater levels (0.28 € m^−3^ versus 0.06 € m^−3^). Whilst in the Netherlands, the reduction in cost was ten-fold (European Commission, 2002). Furthermore, capture and removal of nitrogen on-farm could allow simple and fast recycling of nutrients.

One proposed strategy for soil remediation is the use of carbon-based sorbents due to their physiochemical properties allowing sorption of many types of pollutant, and their methods of production being relatively simple and cheap (Cornelissen et al., 2005). One type, biochar, has received a great deal of research focus over the past two decades, and has been commercialised by many companies, primarily for use as a soil amendment in agriculture (Gabhane et al., 2020).

For the purposes of soil remediation, the advantages of using biochar stem from the low-cost feedstock (many forms of agricultural/industrial/domestic biomass residues can be used), its sorption capacity (typically similar to activated carbon, a widely-used sorbent) that can be tailored towards different pollutants, and the ease of application to soils, something that other remediation strategies often fail to achieve, such as permeable reactive barriers (PRBs) (European Commission, 2009). Also, biochar can be easily modified during its production to have additional properties relevant to its application, including magnetisation via iron oxide impregnation on its surface (Thines et al., 2017). Magnetic biochar can be defined as biochar that has been impregnated with magnetic particles through various methods (described in Section 2.5), rendering it with magnetic properties. Such particles include magnetite, maghemite, hematite, and zero-valent iron, and often exist as nanoparticles bound to the surface of biochar or contained within pores. Some unmodified biochars may also be classed as magnetic, for example where feedstocks contain high concentrations of iron which successfully form magnetic particles through chemical changes induced by pyrolysis (Rodriguez Alberto et al., 2019; Wurzer and Mašek, 2021). There is, therefore, a large variety of physical and chemical properties of magnetic biochars, as affected by the magnetic particles present and the mode of impregnation, which subsequently leads to variability in mechanisms and capacity for sorption of nitrogenous pollutants. Magnetic biochar may vastly widen the scope of applications of biochar due to its removability from media by magnetic separation (Feng et al., 2016; Li et al., 2020b; Xin et al., 2017). In the context of soil remediation, this presents opportunities for sorption and, critically, the subsequent removal of pollutants (such as nitrogen), rather than just immobilisation or reducing bioavailability. Not only would this minimise the risk of pollutants being released back into a bioavailable or leachable form, but it means pollutants and biochar could be recycled. In doing so, the system could operate within a circular economy, reducing the amount of fertiliser that needs to be brought onto the farm from industrial suppliers. This would, therefore, minimise the economic and environmental burden generated by agrochemical production and transport, which can be significant as shown in various Life Cycle Assessments of N fertiliser use (Charles et al., 2006; Hasler et al., 2015; Wang et al., 2007), while simultaneously limiting the amount of waste produced. Also, removal of magnetic biochar reduces the likelihood of negative impacts on the environment, such as ecotoxicity towards organisms that could be caused by re-release of sorbed pollutants (Han et al., 2017).

**Fig. 1:**
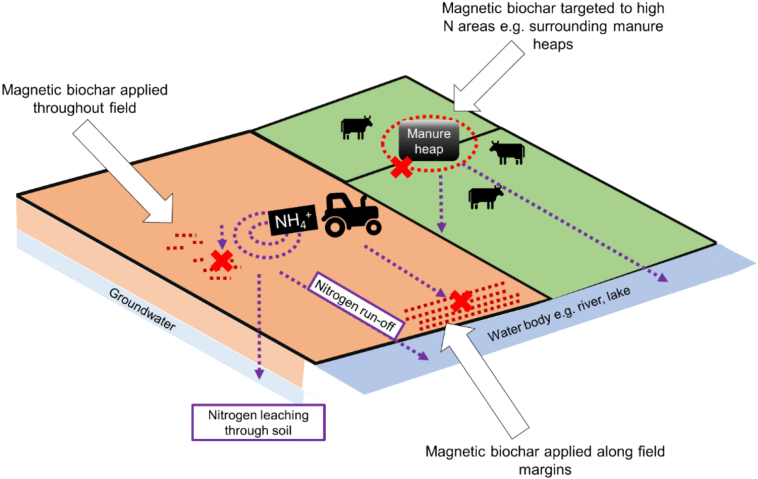
Potential options for optimal spatial targeting of magnetic biochar on farmland for removal of excess ammonium.

The main question is, at what point would it be most effective to capture and remove nitrogen? This needs to be considered in relation to typical agricultural systems (for example, times and methods of harvest and ploughing), but also in terms of the nitrogen cycle. Spatial considerations also apply here, as a targeted approach could maximise overall efficiency of nitrogen. The approach to location could be to prevent nitrogen escape from manure heaps, to remove N from the whole-field, or to target specific areas, such as buffer strips or field margins (Fig. 1). The latter may be the most cost-effective, as it allows plants to access their nitrogen requirements, but any excess that leaves the field via run-off could be captured in a relatively small area. This also means magnetic biochar addition and removal could be unaffected by other farm activities as it would be in a relatively unworked area of the field. However, this may not be as effective at preventing groundwater contamination as leaching occurs vertically in soil.

The nitrogenous compound to be captured is also important. The two main candidates are ammonium (NH_4_^+^) and its nitrified (indirect) product, nitrate (NO_3_^−^). From a pollution perspective, nitrate is the problematic compound as it causes the environmental problems previously mentioned. However, capturing an earlier stage in the nitrogen cycle, ammonium, could be more efficient as it would minimise formation of nitrates. Yang et al. (2015) demonstrated that biochar could efficiently sorb ammonium and simultaneously limit its biotransformation into nitrate. Furthermore, it may be more effectively sorbed by biochar (which is generally negatively charged) as it is a positive ion (Zhang et al., 2020), but this remains to be proven for magnetic biochar (Fidel et al., 2018). The nitrogenous target compound to be captured is largely dependent on where and when sorption occurs – the more detached in time and space from the application of nitrogen to soil, the more likely it will be that nitrates will be a prevalent pollutant, but sorption near to the source would be better targeted towards ammonium.

However, to become a viable remediation strategy, the costs of modifying biochar and removing it from soil must be demonstrated to be compensated for by its potential to significantly reduce leaching and run-off and to improve nitrogen management on farms. Magnetisation of biochar can incur high energy and resource demands, inflating the cost of production, which may already be high due to processing and transporting of residues for pyrolysis. Furthermore, the effects of biochar on soil ecosystems are variable, such as increasing microbial diversity in some experiments while decreasing it in others (Jenkins et al., 2017; Malev et al., 2016; Marks et al., 2014; Noyce et al., 2015); therefore, the proposed use of magnetic biochar must be proven to have minimal negative effects on the soil biome. Due to the fundamental role of the nitrogen cycle in plant, microbe and soil health, the potential ecological effects arising from using biochar to remove nitrogen from soil are complex.

The opportunity for biochar as a carrier for agrochemicals has been reviewed by Sashidhar et al. (2020), who suggest that ‘tuneable’ biochar could be used as a highly efficient controlled release fertiliser, using recent publications on sorption mechanisms and biochar-soil interactions as evidence. This work presented herein will therefore critically consider the evidence for magnetic biochar as a soil remediation strategy by evaluating the rationales, methodologies and interpretations of results in existing research, while establishing the steps required to determine the viability of magnetic biochar as a field-scale soil amendment that is safe, effective, environmentally friendly and sustainable. The focus will be on nitrogen pollution from UK agriculture, as this is established as a serious pollutant of continuing concern that has also shown potential for sorption by biochar in previous work. This work goes further by considering the ways in which biochar could be loaded with nitrogenous compounds in the context of a simultaneous remediation strategy, specifically by utilising magnetic biochar. As a result, a novel soil remediation method is put forward, further evidence is introduced, and the interdisciplinary requirements of the proposed system are made clear.

## 2 Magnetic biochar synthesis for ammonium removal from soil

### 2.1 Overview

**Fig. 2:**
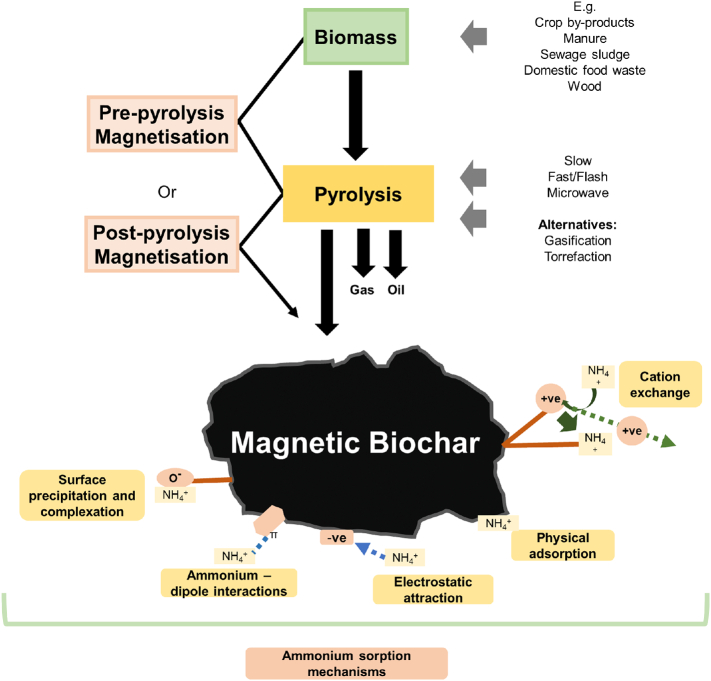
Magnetic biochar production overview with non-exhaustive examples of possible feedstocks and pyrolysis methods, and the range of ammonium sorption mechanisms that may occur. Different combinations of these variables can lead to production of vastly different magnetic biochar types, which can subsequently affect ammonium sorption mechanisms and capacity.

Biochar is produced by the thermal decomposition of organic matter in anoxic or almost-anoxic conditions. The process is broadly termed ‘pyrolysis’, but there are a variety of conditions under which pyrolysis can occur, in addition to a range of feedstocks and additional treatments that can be used. Variability in any of these conditions, treatments or feedstocks can have significant effects on the physical and/or chemical properties of the biochar product (for example porosity, surface area, available functional groups and pH), leading to enhanced or reduced capabilities for sorption or other applications (Fig. 2). Understanding these conditions is therefore critical in developing biochar for ammonium sorption. While previous reviews have discussed the effects of pyrolysis conditions on biochar material properties (Hassan et al., 2020; Huang et al., 2016b; Ippolito et al., 2020; Li et al., 2016a, Li et al., 2019) and the methods of biochar magnetisation, and its use for pollutant sorption (Thines et al., 2017; Li et al., 2020a), this is the first to assess the plausibility of magnetic biochar for ammonium sorption in soil, while considering technical and logistical challenges in the wider remediation landscape.

### 2.2 Feedstock selection for ammonium sorption in soil

In selecting feedstock for biochar to be used in soil ammonium sorption, three key factors must be considered. Firstly, the physical and chemical properties of biomass, as these translate into the properties of biochar. When using biochar for ammonium sorption, the abundance and nature of surface functional groups are critical properties, as functional groups are known to play a role in ion exchange, complexation, precipitation, electrostatic attraction and other mechanisms (Takaya et al., 2016a; Safaei Khorram et al., 2016; Mosa et al., 2018; Hu et al., 2020) (Fig. 2). For example, higher proportions of calcium and magnesium ions in the feedstock can enhance cation exchange capacity (CEC) in the biochar, improving overall sorption of cations like ammonium from the environment (Zhao et al., 2013). Other features relevant to ammonium sorption, such as porosity and surface area, can be influenced by feedstock too, which may be due to the content of lignin, hemicellulose, and cellulose in the feedstock (Li et al., 2014; Zhao et al., 2014). Pores can originate from cell walls in the vascular system of plant-derived biomass, therefore cell wall composition will affect thermal decomposition and the retained porous structure (Lee et al., 2013).

Previous reviews have comprehensively investigated the main trends between feedstock type and biochar properties, but the results of these can be summarised for clarity. Feedstocks can be broadly classed as either wood-based, crop-based, or manure/biosolid-based. Meta-analyses by Ippolito et al. (2020), Li et al. (2019), and Hassan et al. (2020) all conclude that generally, wood-based biochars had the greatest specific surface areas while crop-derived biochars had an overall greater CEC, suggesting greater surface functionality. The third main type, manures/biosolids, had low surface area but high CEC. Li et al. (2019) also showed that biosolid/manure-based biochar had the greatest N retention in soils, suggesting a higher proportion of functional groups able to interact with nitrogenous compounds. Based on these conclusions, tailoring biochar production for ammonium sorption would consider main sorption mechanisms (for example, exchange with surface cations), and selection of appropriate feedstock – in this case, manure/biosolid-based biochar may provide the highest sorption capacity.

Secondly, availability of feedstock is important when working towards field-scale application of biochar for remediation purposes. Feedstocks from agricultural residues (for example corn stalks (Wang et al., 2017), empty fruit bunches (Mubarak et al., 2014), and sugar beet tailings (Zhang et al., 2012)), industrial waste (for example, paper mill sludge (Devi and Saroha, 2014) and spent brewers grain (Zhang and Wang, 2016)) and domestic waste (for example, municipal solid waste (Takaya et al., 2016b) and sewage sludge (Yang et al., 2018)) have all been used to successfully prepare biochar for sorption of a range of pollutants. In these instances, the production of biochar is typically on a small scale (producing less than a kilogram of biochar) for laboratory experiments, but large pyrolysis units are capable of processing thousands of kilograms of biomass per hour (Khodaei et al., 2019) and could therefore provide technical solutions to large-scale manufacture. However, availability of biomass needs to be considered, as some feedstock types, such as agricultural residues, will fluctuate in availability due to season, market prices and unpredictable weather events, such as droughts or flooding. A mixed feedstock approach, using material from a range of sectors, could be one potential way to mitigate against this risk. However, a mixed-feedstock approach could lead to production of highly variable biochar (for example, its sorption capacity), creating difficulties for ‘tailor made’ biochar engineering.

Thirdly, in order to meet wider sustainability requirements, such as a low carbon footprint of the biochar product, intelligent feedstock selection is critical to ensure that the feedstock does not cause issues elsewhere, such as deforestation of fragile habitats. Furthermore, if feedstock needs to be transported great distances for production, the carbon emissions of the process may be increased. Also, the presence of contaminants such as heavy metals in the feedstock (Zhao et al., 2018), or the process of pyrolysis producing toxic compounds like polycyclic aromatic hydrocarbons (PAHs) and dioxins (Hale et al., 2012), may lead to biochar releasing pollutants into soil, posing a risk to human health. It is therefore critical that feedstock-pyrolysis interactions are understood before application of biochar to the environment.

### 2.3 Microwave pyrolysis for magnetic biochar synthesis

**Fig. 3:**
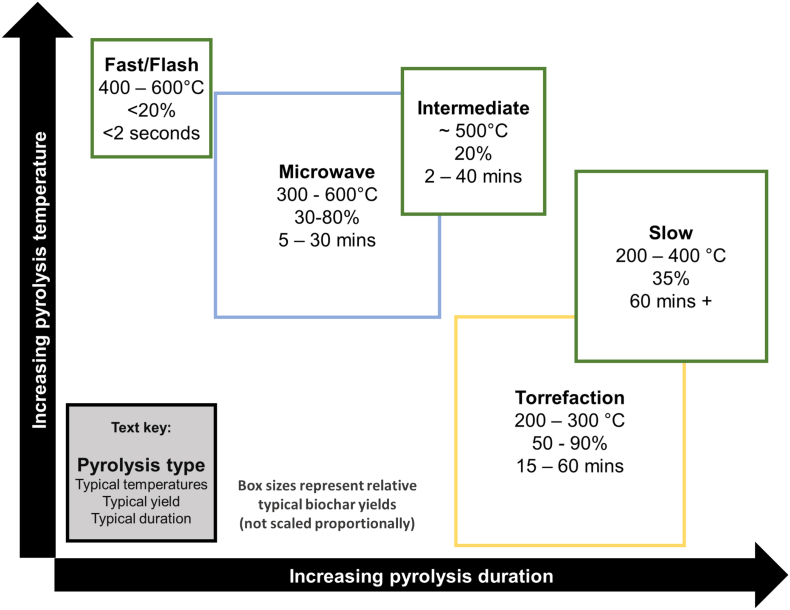
Comparison of main pyrolysis methods used in biochar production. Microwave pyrolysis can produce relatively higher yields than other methods, while requiring only a short heating duration and low energy inputs. However, other factors not shown in this simplified graphic, such as equipment costs, transport between sites, processing requirements, and influences on morphological and chemical characteristics of the products, are also important considerations.

Pyrolysis can occur by different methods. The varying rates and means of heating biomass (Fig. 3) lead to different patterns of thermal decomposition, meaning different pyrolysis methods can affect biochar yield and properties. Furthermore, balancing energy-efficiency with biochar yield is a key consideration for biochar synthesis. As a result of this, there is growing interest in microwave pyrolysis, as this method is relatively energetically efficient and still produces a comparatively high yield of biochar (Abas and Ani, 2014). It has been proposed as an effective, low emission, low energy and selective alternative to ‘conventional’ methods and has been used experimentally to produce biochar for sorption applications (Abas and Ani, 2014; Thines et al., 2017) (Table 1). Previous reviews, such as those by Li et al. (2016a) and Huang et al. (2016b), have provided comprehensive overviews of the range of material properties rendered by microwave pyrolysis into biochar. However, a lack of methodological information in many studies makes quantification of energy-efficiency difficult (for example, the rate of energy transfer to a given mass of biochar).

**Table 1:** Pyrolysis conditions of biochars produced from UK-available feedstocks through the microwave (MW) method.

Biomass Feedstock	a Pyrolysis Conditions	b MW Susceptor?	Biochar Yield	Application	Source
Biosolids	600 W 10 mins –	Y	85.5%	MW optimisation	(Antunes et al., 2018)
Cellulose	300 W – –	Y	~ 35–78%	MW optimisation	(Al Shra’Ah and Helleur, 2014)
Corn Stover	– 45 mins 600 °C	Y	N/A	Supercapacitors	(Jin et al., 2014)
Corn Stover	– 18 mins 650 °C	N	N/A	Phosphorus Sorption	(Chintala et al., 2014)
Maple Wood	300 W – 290/330 °C	N	N/A	Characterisation	(Dutta et al., 2015)
Municipal Solid Waste	2000 W – 550 °C	N	~ 78–83%	Yield optimisation	(Li et al., 2018)
Rapeseed Shell	600 W – –	Y	19.98–41.2%	MW optimisation	(Fan et al., 2019)
Sewage Sludge	750 W – 450–600 °C	Y	~ 50 to 72%	MW optimisation	(Xie et al., 2014)
Sewage Sludge	1200 W 10 mins –	Y	N/A	MW optimisation	(Zhang et al., 2018)
Spent Mushroom Substrate	– 35 mins –	Y	30–36%	Fertiliser	(Lam et al., 2019)
Spruce Pellets	2000/3000 W, 30/60/120 mins, 150–250 °C	Y	32.4/26.2%	Characterisation	(Nhuchhen et al., 2018)
Straw Pellets	1200 W – –	N	33.7%	Characterisation	(Mašek et al., 2013)
Wheat Straw	900 W 30 mins –	N	N/A	As(V) & Methylene blue sorption	(Zubrik et al., 2018)
Willow	300 W – –	N	27.3%	MW optimisation	(Gronnow et al., 2013)
Willow	1200 W – –	N	27.3%	Characterisation	(Mašek et al., 2013)
Wood Biomass	–	N	45.2%	Gasification	(Wu et al., 2015)

a Pyrolysis conditions – MW power (w), heating time (mins) and temperature (°c).

b Use of microwave susceptor – yes (Y) or no (N).

In addition to these potential economic and logistical benefits of microwave pyrolysis, this method is of particular interest in this review because it may render a higher quality magnetic biochar product. Conventional methods, where biomass is heated from the outside inwards, can lead to overheating of the exterior, which in turn causes surface cracking, potentially affecting pore formation, stability and magnetic particle deposition, whereas the volumetric heating mechanism of microwave pyrolysis prevents this effect (Salema and Ani, 2011). In addition, Zhu et al. (2014) observed that magnetic biochar produced from cotton fabric by microwave pyrolysis had magnetic particles with a more uniform size and less agglomeration than conventionally pyrolysed magnetic biochar. This could reduce the amount of pore blocking and shedding of magnetic particles, improving efficiency as a sorbent material. However, its potential for large scale biochar production needs to be properly evaluated if it is to become a viable alternative to producing biochar for remediation purposes. At its most simplified level, conventional pyrolysis can operate using basic kilns or open pits and has therefore been used successfully for on-farm production (many examples of these systems can be found in rural communities in Africa, India, and south-east Asia). These structures can be large and used in remote areas, as they can be powered by conventional energy sources, such as combustion of biomass or fossil fuels (although greenhouse gas emissions remain an issue under this method). Microwave pyrolysis units are powered by electricity and therefore may add a substantial layer of complexity to scaling-up to on-farm production where access to electrical power is limited. Potential sustainable solutions to this could involve a combination of microwave pyrolysis units with renewable electrical energy production and/or re-using pyrolysis products such as oils and gases for conversion to electrical energy. However, technological challenges also remain in adapting microwave pyrolysis units for processing large quantities of biomass. Most of the research so far has used adapted domestic microwave ovens or small custom-made devices, but research using much larger units is needed to investigate effects on product quality and overall operation efficiency and safety.

### 2.4 Magnetic biochar overview

The relatively simple procedure of magnetising sorbents was pioneered by Šafařík and colleagues in the 1990s through co-precipitation of iron oxides onto a range of sorbents for removal of dyes from solution (Safařik, 1991; Šafařík et al., 1995, Šafařík et al., 1997). The idea of combining magnetic particles with novel, easily produced sorbents soon followed, with studies using activated carbon showing further promise for remediation (Oliveira et al., 2002; Zhang et al., 2007). The primary intention for magnetisation was to enable simple, cheap, and efficient separation of analytes and pollutants from aqueous solution. As biochar studies grew exponentially through the 2010s, various researchers began to magnetise biochar. Chen et al., 2011a, Chen et al., 2011b were among the first, developing a ‘one-step’ method of co-precipitation and pyrolysis from which magnetic biochar was produced with good magnetic properties and increased sorption of organic contaminants and phosphate. A secondary potential benefit of magnetisation is therefore now also a factor to be considered – the enhanced sorption abilities of magnetic biochar over non-magnetic (but otherwise equivalent) biochar. Moreover, research is now uncovering links between iron speciation and pollutant removal mechanisms; Xu et al. (2021), for example, found that the carbon structures present in production interact with iron to form different iron species, allowing magnetic biochar to be tailor-made for enhanced arsenic removal via immobilisation. However, the magnetisation process may be seen as too costly or complex compared to other remediation technologies (Vikrant et al., 2018), so as an emerging strategy, research needs to include a focus on scalability and cost-effectiveness. Synthesis of magnetic biochar so far has been at relatively small scales, producing less than 0.1 kg – a suitable amount for characterisation and sorption experiments, but not a usable quantity for field applications.

### 2.5 Methods of magnetisation

Various techniques can be used to magnetise biochar, but two main methods are used – co-precipitation and iron solution pre-treatment. The former is generally performed after pyrolysis, whereby iron oxides are precipitated onto the biochar surface under alkaline conditions (hydroxide ions (OH^−^) react with iron ions to form intermediate products which then react to form magnetite). The latter tends to occur before pyrolysis, whereby the feedstock is first saturated with an iron solution for a short period of time, which then form magnetic particles during pyrolysis (Safarik et al., 2012). Interestingly, the need for co-precipitation or iron solution pre-treatment has been challenged by a novel approach demonstrated by Rodriguez Alberto et al. (2019), using the digestate from an anaerobic digestion process fed by cow manure and industrial food wastes, followed by pyrolysis to produce magnetic biochar. Similarly, other iron rich waste streams have been proposed as sustainable reagents for magnetisation, with Wurzer and Mašek (2021) showing that ochre (mining waste) could be effectively used to form magnetite/maghemite impregnated biochar with increased adsorption capacity for caffeine and fluconazole. Rodriguez Alberto et al. (2019) attributed the formation of magnetite particles on their biochar (approximately 3.1% Fe) to the high iron content of the solid digestate combined with the thermochemical processing parameters. For comparison, Wan et al. (2020) found their biochar, produced from cedar sawdust, contained 0.08% Fe, but this increased to 27.9–48.7% when iron was introduced by co-precipitation at a preparation stage. Using the same method and eucalyptus woody debris (EB) and pig manure (PB), Wu et al. (2021) found increases from 0.013% to 23% Fe (EB) and 0.021% to 19% (PB). Also, Zeng et al. (2021) activated biochar using different concentrations of FeCl_3_ and found levels of iron in the product increased from 0.04% Fe in unmodified biochar to 7.29% Fe in the modified biochar with the lowest impregnation mass ratio of FeCl_3_ to biochar (0.5). Importantly, they found that the iron concentration of biochar affected the subsequent properties and adsorption capacity. Across other studies, the type of magnetic particles formed varied not only by method, but with pyrolysis conditions and feedstock. The reaction itself, or simply the addition of magnetic nanoparticles, can alter the surface of biochar and therefore have an effect on properties such as sorption capacity. Analytical techniques, such as SEM, have shown magnetic biochar to have structural differences compared to unmodified biochar which may affect sorption. For example, Liao et al. (2018) found pores to be blocked by magnetic particles, while Saleh et al. (2016) found the surface to exhibit a rougher texture, which could provide more sorption sites. Table 2 summarises the range of methods and products that have been used experimentally, demonstrating the scope of applications permitted by different magnetic biochars.

**Table 2:** Magnetisation methods for different biochars and consequential magnetic properties and applications in water.

Method	Pre/post pyrolysis	Medium	Conditions	Biomass	Magnetic phase	Saturation magnetisation	Application	Source
Co-precipitation	Pre	FeCl_2_ FeCl_3_ (1:1)	pH 10, 30 mins, stirring	Orange peel	Fe_3_O_4_ (magnetite)	N/A	Phosphate & organic removal	(Chen et al., 2011a)
Iron solution treatment	Pre	Fe(NO_3_)_3_ Co(NO_3_)_2_	Ethanol, 15 mins, stirring	Pine bark	CoFe_2_O_4_ (Cobalt ferrite)	N/A	Pb^2+^ & Cd^2+^ removal	(Reddy and Lee, 2014)
Iron solution Treatment (+ Surfactant + reducing agent (Zero Valent Iron))	Post	FeSO_4_ CTMB NaBH_4_	30 mins, Vigorous stirring	Paper mill sludge	Fe^0^ (Zero valent Iron)	N/A	PCP removal	(Devi and Saroha, 2014)
Iron solution treatment	Post	Fe(acac)_3_	30 mins, 180 °C, Vigorous stirring N_2_ protection	Rice hull	Fe_3_O_4_	13.6 emu/g	Pb^2+^ removal	(Yan et al., 2014)
Co-precipitation	Post	FeCl_3_ FeSO_4_ (2:1)	pH 10–11, 60 mins, stirring	Eucalyptus leaves	Fe_3_O_4_	16.0 emu/g	Cr^6+^ removal	(Wang et al., 2014)
Co-precipitation	Post	FeCl_3_ FeSO_4_ (2:1)	pH 10, 60 mins, stirring	Mixed wood chips	Fe_3_O_4_	N/A	Phenanthrene & phenol sorption	(Han et al., 2015)
Hematite treatment	Pre	Hematite mineral solution	120 mins, mixed	Pine wood	ɣ-Fe_2_O_3_ (maghemite)	N/A	As^5+^ sorption	(Wang et al., 2015a)
Iron solution treatment	Pre	FeCl_3_	24 h, Immersed	*Eichhornia crassipes*	ɣ-Fe_2_O_3_	11.6 emu/g	Cr^6+^ removal	(Zhang et al., 2015)
Iron solution treatment	Pre	FeCl_3_	60 mins, Stirring, 70 °C	Peanut hull	ɣ-Fe_2_O_3_	36.79 emu/g (at 650 °C pyrolysis)	Cr^6+^ removal	(Han et al., 2016)
Ball milling	Post	Fe/ α-Fe_2_O_3_/ or Fe_3_O_4_	Ball milled, 6 h, 550 rpm	Nut shells	Fe_3_O_4_	19.0 emu/g	Carbamazepine & tetracycline sorption	(Shan et al., 2016)
Co-precipitation	Post	FeCl_2_ FeCl_3_ (1:1)	NaOH, 30 mins, stirring	Palm kernel shell	Fe_3_O_4_	N/A	4-nitrotoluene removal	(Saleh et al., 2016)
Iron solution treatment	Pre	Fe_3_O_4_	30 °C, 200 rpm	Sugarcane bagasse	Fe_3_O_4_	6.138 emu/g	Cd^2+^	(Noraini et al., 2016)
Oxidative hydrolysis	Post	FeCl_2_ KOH KNO_3_	90 °C N_2_ protection	*Pinus radiata* sawdust	Fe_3_O_4_	47.8 emu/g	Sulfameth -oxazole removal	(Reguyal et al., 2017)
Co-precipitation	Post	FeCl_2_ FeCl_3_	Stirring, 120 mins, Additional pyrolysis	Rattan	Fe_3_O_4_ FeO α-Fe	27.11 emu/g (max.)	Properties	(Hu et al., 2017)
Co-precipitation	Pre	FeCl_3_ MgCl_2_ (2:1)	pH 10, stirring, 60 °C, 4 h	*Undaria pinnatifida* roots	MgFe_2_O_4_	52.48 emu/g.	Phosphate Sorption	(Jung et al., 2017)
Iron solution treatment	Pre	Fe(NO_3_)_3_	80 °C, 120 mins	Corn husk	Fe_3_O_4_	14.87 emu/g	Paraquat removal	(Damdib et al., 2019)
Co-precipitation	Post	FeCl_3_ FeSO_4_	pH 10 N_2_ protection	Cellulose	Fe_3_O_4_	10.7 emu/g	Plastic sorption	(Tong et al., 2020)
Co-precipitation	Post	FeCl_3_ FeSO_4_	pH 10–11 Agitation, 60 mins	Rice husk	Uncharacterised	N/A	Dye sorption	(Trinh et al., 2019)
Pyrolysis of Anaerobic Digestate	Pre	None	None	Digestate from manure/food waste	Fe_3_O_4_	N/A	Characterisation	(Rodriguez Alberto et al., 2019)

Most studies focus on the characteristics of the biochar rather than on the magnetic particles, meaning magnetism could be improved with further research. However, Reguyal et al. (2017) developed methods to more precisely select the magnetic particles to be deposited on biochar, with the aim of reducing proportions of lower and non-magnetic phases of iron oxides and iron hydroxides and increasing magnetite deposition. By using oxidative hydrolysis of FeCl_2_ in alkaline media, undesirable phases were prevented from being formed, producing biochar with very high saturation magnetisation (47.8 emu/g) compared to other methods.

However, characterisation of magnetic biochar by Brunauer–Emmett–Teller (BET) surface area analysis, X-ray diffraction (XRD), scanning electron microscopy (SEM) and magnetometry is needed alongside sorption experiments to determine antagonistic effects of magnetic particles, such as the blocking of pores, which could reduce surface area and affect sorption (Fu et al., 2011; Li et al., 2017a; Liao et al., 2018). Comparing magnetic biochar with non-magnetic biochar should therefore be fundamental as this can help elucidate the effects of magnetisation; for example, Sani et al. (2016) showed that magnetite impregnation did not detrimentally affect ibuprofen and diclofenac sorption. However, sorption of some pollutants (for example, ammonium ions) appears to be mostly dependant on the presence of surface functional groups, such as -COOH, -OH, C=C, C=O and -CH_2_-, permitting mechanisms like CEC (Cai et al., 2016; Cui et al., 2016; Takaya et al., 2016a; Tian et al., 2016; Hu et al., 2020), so the method of magnetisation clearly needs to be designed in a tailor-made approach towards the pollutant.

### 2.6 Magnetised biochar cost-effectiveness and scalability

The main costs associated with biochar production are feedstock, treatment(s), pyrolysis, storage and transport. Additional costs for magnetisation of biochar are chemicals and processing. Finally, additional costs for magnetic biochar extraction from soil (here onwards referred to as MBES) are separation equipment purchase/hire, energy and additional processing of collected magnetic biochar. At all stages, there is potential to minimise costs (Table 3).

**Table 3:** Potential expenses to be encountered and cost-minimisation strategies in the proposed system.

Process	Expense	Example(s)	Potential cost-minimisation strategy
Biochar production	Feedstock	Biomass	■ Use of low-value agricultural by-products or waste.
	Treatment(s)	Washing, modification	■ Simplified ‘one-step’ modification/pyrolysis methods (Chen et al., 2011a, Chen et al., 2011b).
	Pyrolysis	Energy, labour, quality control, equipment	■ Microwave pyrolysis for lower-energy inputs ■ Agricultural co-operatives to share costs
	Storage/transport	Safe storage of flammable particulate matter, off-farm transport	■ Production and storage at/near farm to be used on.
Magnetisation	Chemicals	Iron solution, NaOH	■ Optimisation of methodology to maximise Iron solution-to-magnetic particle conversion efficiency.
	Processing	Stirring, shaking, heating, N_2_ supply, labour	■ *Re*-use of magnetic biochar to reduce frequency of production.
MBES	Separation equipment	Purchase/hire of specially designed technology	■ Agricultural co-operatives to share costs. ■ Subsidies (from e.g. government agencies)
	Energy	Fuel for vehicle, separator rotation	■ Maximising time between separation runs (without jeopardising efficient pollutant removal). ■ Mechanical innovations to utilise pulling forces of vehicle.
	Additional processing	Washing, pollutant desorption	■ Recycling as many components as possible, such as desorption medium and biochar.
Quality Control	Testing	Potential biochar derived pollutants e.g. Fe, PAH.	■ Adherence to quantified biochar standards e.g. International Biochar Initiative. Use IBI certified products.

Further aspects that need to be considered in using MBES at field-scale are the potential environmental effects. Addition of biochar has been shown to have a wide range of positive and negative effects on soil ecosystems, but additional interactions when using magnetic biochar could further complicate the issue. For example, ferromagnetic particles could become dislodged from biochar in soil, possibly altering soil chemistry. Although this may not have a negative effect on soil ecology (for example, Rui et al. (2016) showed that maghemite nanoparticles could be used as an Fe fertiliser to improve plant growth after they were adsorbed onto the sandy soil), the use of synthetic nanoparticles in the environment is an area which should be approached with caution (Javed et al., 2019). Because of this, some studies have quantified the levels of enzymatic activity and bacterial community size/composition after addition of metal oxide nanoparticles. For example, zinc oxide nanoparticles exhibited strong effects on enzymatic activity and bacterial communities (such as reduced total bacterial population size), while magnetite nanoparticles exhibited only mild effects (You et al., 2018). Interestingly, the effect differed across soil types, adding to the complexity of the issue.

Finally, while the effect of long-term addition of biochar to soil is somewhat understood, due to the novelty of MBES the effects of removing pollutant-laden biochar from soil are unknown. Since soil chemistry changes after biochar addition, it is not unreasonable to speculate that changes will occur when it is removed, and these may not be a simple reversal of the initial changes induced on biochar addition. In addition, it is reasonable to expect that the physical effect of removing biochar from soil could induce complex changes. For example, microorganisms have been shown to colonise biochar after its addition to soil (Ascough et al., 2010; Hammer et al., 2014), potentially to obtain nutrients, or to utilise the habitat that the porous structure of biochar offers. Subsequent removal could therefore extract components of the soil ecosystem. However, the extent to which organisms colonise biochar over different time periods, and whether removal could significantly impact on soil ecology, is still unclear and is likely to vary greatly depending on biochar characteristics. Other physical changes that should also be considered are soil-water-biochar interactions. Biochar has been shown experimentally, for example, to increase the water holding capacity of loamy sandy soils (as much as double in a study by Yu et al. (2013), although this was a short-term laboratory study using a relatively high biochar amendment rate of 9% so may not accurately reflect field conditions). The removal of biochar could therefore be predicted to involve removal of soil water. Fluctuations in water content in soil therefore need investigation during biochar addition and removal to ensure the physical structure of soil is not adversely affected.

### 2.7 Ammonium sorption by biochar

Multiple studies have considered the ammonium sorption capacity of biochar (Table 4) in liquid media. Many conclude that the dominant mechanisms of sorption stem from the biochar functional groups rather than the surface area and porosity. Cai et al. (2016) determined that despite lower pyrolysis temperatures producing biochar of lower surface area and porosity, low temperature biochars (200 °C) retained oxygen functional groups that improved sorption of ammonium via electrostatic attraction and hydrogen bonding. Fidel et al. (2018) concurred with this, showing higher sorption at the lower pyrolysis temperature (400 °C), as did Gao et al. (2015) (low pyrolysis temperature of 300 °C), again attributing the phenomenon to the retention of oxygen containing groups. Tian et al. (2016) found that CEC was enhanced at a lower pyrolysis temperature (400 °C), while surface area was enhanced at a higher pyrolysis temperature (500 °C), but CEC was the dominant mechanism for ammonium sorption and so the lower pyrolysis temperature was again found to be optimal. Similarly, Zhang and Wang (2016), demonstrated yield increases with decreasing pyrolysis temperature, another benefit. Finally, Hu et al. (2020) compared a range of pyrolysis conditions, including four different temperatures up to 600 °C, and found that the lowest temperature (300 °C) correlated with the highest sorption capacity. In addition, they used Fourier transform infrared (FTIR) analysis to show that at increasing temperatures, peaks corresponding to carboxyl and hydroxide groups declined, again strengthening the evidence that low pyrolysis temperatures improve ammonium sorption due to the retention of oxygen-containing groups. The results of Xu et al. (2019) concur with this, finding an increasing ratio of oxygen to carbon as pyrolysis temperature decreased, resulting in greater aromaticity.

**Table 4:** Surface areas and ammonium sorption by different biochars.

Biochar	sBET Surface Area (m^2^ g^−1^)	a NH_4_ sorption Q_max_ (mg g^−1^)	Notes	Source
Wood	37.56	0.15	CEC was dominant mechanism	(Tian et al., 2016)
Bamboo	330	0.852	Primary mechanism – ion exchange	(Ding et al., 2010)
Corn stover	No data	1.1	pH 7–7.5 was optimal	(Fidel et al., 2018)
Poultry litter	15.43	1.3	CEC was dominant mechanism	(Tian et al., 2016)
Digested sludge	20.86	1.4	450 °C biochar performed best	(Tang et al., 2019)
*Phragmites communis*	3.5	3.2	Higher sorption attributed to zeta-potential and C/H ratio	(Xu et al., 2019)
Sawdust	378.7	3.3	Higher sorption attributed to zeta-potential and C/H ratio	(Xu et al., 2019)
Rice straw	34	4.1	Higher sorption attributed to zeta-potential and C/H ratio	(Xu et al., 2019)
Rice husk	179	4.7	Over 2× the sorption capacity of NO_3_	(Pratiwi et al., 2016)
Mixed hardwood	No data	5.29	18% of total amount removed	(Sarkhot et al., 2013)
Orange peel	0.54	5.6	Low temp biochar (300 °C)	(Hu et al., 2020)
Oak sawdust	1.57	10.1	Lanthanum-modified biochar	(Wang et al., 2015c)
*Canna indica*	7	13.35	Lower sorption than of cadmium ions	(Cui et al., 2016)
Wheat straw	4	15.5	No NO_3_ adsorbed under same conditions	(Gai et al., 2014)
*Thalia dealbata*	223.08	17.6	Phosphate sorption also maximised	(Zeng et al., 2013)
Sugarcane leaves	27.9–218.9	22	MgO-modified biochar. Sorption the same for all SAs.	(Li et al., 2017b)
Corn cob	0.051	22.6	Modified by soaking in HNO3 and NaOH	(Vu et al., 2017)
Rice husk	11	71.94	–	(Kizito et al., 2015)
Hardwood	147	114.2	Low SA chars also performed well.	(Kizito et al., 2016)
Wood	273.6	133.33	–	(Kizito et al., 2015)
Presscake	2.5	136.2	Phosphate sorption occurred, but at lower capacity	(Takaya et al., 2016a)
Corn cobs	No data	243.3	Low-temperature biochars showed relatively fast sorption kinetics	(Gao et al., 2015)

a Maximum sorption capacity as calculated by adsorption isotherm.

It is reasonable, therefore, to conclude that in designing biochar for ammonium sorption, the primary focus should be on the creation/retention of reactive functional groups, rather than maximising overall surface area. While the evidence from conventional pyrolysis methods strongly suggests a trend of decreasing surface functional group density as temperature increases across the temperature range of 200 to 600 °C, whether the same correlation occurs under microwave pyrolysis is unclear. However, Abdelsayed et al. (2018) provided some evidence that there may be notable differences between surface functional groups on conventional- and microwave-produced biochar. At a temperature of 550 °C, they observed that coal biochar pyrolysed conventionally contained a significantly higher concentration of functional groups compared to coal biochar pyrolysed through microwave heating. Through comparison with FTIR spectra of biochar produced at 900 °C, they observed that the absorption spectrum of the microwave pyrolysis biochar (550 °C) was similar to the conventionally pyrolysed biochar (900 °C) and concluded that although the bulk temperature remained at 550 °C, microwave-generated hotspots may reach much higher temperatures, leading to loss of functional groups. This could be due to the dipole of functional groups coupling with the electric field of the microwaves, although this effect is still an area of dispute in the field of microwave chemistry (De La Hoz et al., 2005). If this phenomenon is relevant to all biochars produced by microwave technology, using temperatures lower than those that would be used for conventional pyrolysis may be required to ensure a high density of surface functional groups remain. However, more evidence is required to elucidate the effect of microwave heating on biochar functional groups, as these data come from just one study which used coal as the feedstock, which is likely to be chemically different from other feedstocks.

If magnetic biochar for ammonium sorption can be created simply by using lower pyrolysis temperatures (< 400 °C), and without the long heating duration associated with slow pyrolysis, total process energy demands would decrease per unit of biochar produced, and yield would increase, improving cost-effectiveness and sustainability. For example, Huang et al. (2016a) concluded that microwave pyrolysis (using a single-mode 2.35 GHz microwave device) of a variety of feedstocks required less input energy, over a shorter duration, than conventional pyrolysis to cause the same level of thermochemical decomposition. However, little evidence exists to show how ammonium sorption is affected by addition of magnetic particles to biochar surfaces. Since magnetisation of biochar was reported to improve sorption of nitrate, an anion, in one study (Bombuwala Dewage et al., 2018), if the main mechanism is electrostatic interaction, it may be that cation sorption is less efficient. This hypothesis is further supported by Sun et al. (2019), who observed decreased sorption of cations by magnetic biochar as the impregnation ratio of iron to biochar mass increased from 0.5:1 to 2:1 (which correlated with increased proportions of iron on biochar, determined by ICP-AES analysis), an effect which may be attributed to the increased electrostatic repulsive forces caused by the higher concentration of positively charged iron on the biochar surface. Sorption of phosphate, an anion, has also been shown to be improved after magnetisation, for example Yang et al. (2018) found that magnetic biochar with higher surface iron content adsorbed more phosphate, with FTIR showing that after sorption, Fe-OH groups diminished but P—O groups appeared, which could be from phosphate replacing hydroxide groups via ligand exchange.

Further research therefore needs to elucidate if ammonium sorption is affected by magnetisation of biochar, and if so, what the mechanisms behind this are and how improvements can be made. There may, for example, be a balance to be found, where enough magnetic particles are present to render adequate magnetic properties, but do not greatly disrupt surface functional groups required for ammonium sorption. On the other hand, surface modification may not greatly affect sorption, as found by Li et al. (2017b), where increasing levels of Mg on biochar surface made no significant difference to ammonium sorption. In contrast, a study by (Li et al., 2016b) found that phosphate sorption increased with increasing Mg levels on biochar, suggesting again that anions are more affected by surface modification than cations.

### 2.8 Summary

As biochar research develops, it is becoming increasingly apparent that ‘designer’ biochar (so-called due to it being engineered to be optimised for a particular function, such as sorption of a specific pollutant) is a feasible method of optimisation for particular applications, including pollutant sorption. This can be managed through choice of feedstock, pyrolysis methods and treatment. Fortunately, cheap and easily sourced waste can be used as feedstock (although not without extensive prior analysis of economic, environmental and logistical considerations), new pyrolysis methods such as microwave heating offer potential in low-energy, effective production, and treatments, such as magnetisation, have been proven to be relatively simple and low-cost procedures. While more research is needed to better understand the precise effects of these aspects of biochar synthesis on biochar application, another area of research that is currently deficient in data is the scalability and cost-effectiveness of such procedures to on-farm, large-scale production. To date, magnetic biochar has been used in a variety of laboratory experiments, for example in batch experiments to investigate sorption capacity for pollutants. Although experiments using solutions containing a single pollutant are useful in understanding sorption mechanisms, in order to further develop magnetic biochar research, studies where magnetic biochar is used in wastewater or soils, under realistic environmental conditions and/or sourced from the environment, are needed. Some have used environment-sourced wastewater, but this tends to have been done so in a small-scale laboratory setting, using just small quantities of magnetic biochar (e.g. less than one kilogram). Magnetising biochar at scales ranging from hundreds of kilograms to tonnes presents greater challenges than merely increasing quantities of biomass and chemicals used. To ensure iron solutions are adequately integrated into the feedstock, mixing and heating is required. This would require additional equipment which may be large and expensive and therefore problematic for producers of biochar without access to such resources.

## 3 Recycling of magnetic biochar and ammonium

### 3.1 Pollutant desorption and sorbent material recycling

A major incentive for developing magnetic biochar for soil remediation is the potential to remove pollutants from where they persist at excessive levels and to redistribute them to areas that may benefit from them, supporting fertiliser management and net zero and circular economy initiatives. This is particularly relevant for nitrogenous pollutants, as nitrogen is a vital component of soil and only causes problems when levels are too high and leaching or run-off occurs. There are two main ways redistribution could occur. Firstly, ammonium-loaded biochar could be applied directly to soil, allowing slow-release of nitrogen back into the soil for uptake by plants. This would be a relatively cheap method, but it may be difficult to control and monitor nitrogen release. In addition, magnetic biochar would be a more costly amendment than standard biochar, so the expenditure involved in magnetisation may not be offset by benefits from long-term addition to soil. Also, there is a risk of iron leaching into the soil, which could be detrimental to soil health. Although iron is an important element in soils for plant growth (occurring in a variety of forms, such as Fe^3+^, Fe^2+^ and ferric oxides (Fageria et al., 1990)), high concentrations of available iron may adversely affect plants, although iron toxicity is primarily seen in low pH soils, so alkaline biochars may offset this. In addition, other toxic pollutants like arsenic, cadmium, and lead may be adsorbed by compounds such as iron oxides, inhibiting other remediation strategies. Some studies which included iron leaching experiments after using magnetic biochar in aqueous media, demonstrated a range of effects, from low levels of iron leaching in effluent (e.g. Devi and Saroha (2014) recorded 0.21 mg L^−1^ Fe in the remaining solution after using zero-valent iron biochar in effluent containing pentachlorophenol), to higher, pH dependent levels of iron leaching (e.g. Yi et al. (2020) found leaching increased as pH decreased, with Fe levels of 14.63 mg L^−1^ leached at pH 3, using iron oxide biochar in chromium VI solutions). However, although these studies suggest there may be a chemical effect on iron leaching from magnetic biochar, they do not give an indication as to whether iron may be shed as a result of physical soil processes, such as weather-induced biophysical changes like soil saturation or drought, or movement and processing of soil components by organisms (such as earthworms or plant roots). In addition, it is impossible to replicate the vast range of environmental conditions found in soil with such experiments, but they do, however, provide a way to study effects of isolated chemical changes (in this case, pH).

The second method of recycling nitrogen would involve desorption of ammonium from the magnetic biochar, before re-applying the extracted solution as fertiliser (or at the very least, disposing of it in an environmentally safe manner). Assuming the magnetic biochar retains its high sorption capacity and magnetism, it could then be re-applied to polluted soils, reducing the energy and resource demand of production. The second method is therefore theoretically a more desirable goal, but it is dependent on the cost-effectiveness of separating and recycling nitrogen and magnetic biochar. Some studies have investigated the potential of the first method, while others have investigated desorption of nitrogen and recycling of biochar, but not in great detail.

### 3.2 Spent biochar as a slow-release fertiliser

El Sharkawi et al. (2018) demonstrated that biochar loaded with ammonium phosphate slowly released low levels of nitrogen into soil in the forms of ammonium and nitrate during the 45-day experiment, which corresponded to overall higher residual nitrogen in the soil and greater plant growth compared to artificial ammonium phosphate mineral fertiliser in the same timeframe. The presence of nitrate suggests either conversion of ammonium by nitrification processes in the soil, or its existence as an artefact, but this is not reported. Nitrogen in leachate was also significantly lower in the biochar treatments, implying that nitrogen was either bound to the biochar or taken up by plants. However, the biochar-fertiliser composite was assembled synthetically through reaction with phosphoric acid and ammonia gas, so the quantities and attachment of nitrogenous compounds present may be considerably different from biochar that has obtained ammonium purely through sorption. In this case, for example, the phosphoric acid firstly ‘activates’ the biochar, after which the increased availability of acidic functional groups allows increased binding of ammonium, a reaction also shown experimentally by Ro et al. (2015). In non-activated biochars, the degree of adsorption may be less than activated biochars, or ammonium may be adsorbed by alternative mechanisms. Analytical techniques can determine what attachment mechanisms are used, such as Scanning Electron Microscopy (SEM) combined with Energy Dispersive X-ray Spectroscopy (EDS) to determine if ammonium has formed compounds with other elements bound to biochar, for example magnesium ammonium compounds, as found by Cui et al. (2016). Furthermore, FTIR is an effective way to examine changes to chemical bonds on the biochar surface, allowing inference of binding mechanisms, while cation exchange capacity (CEC) tests allow investigation of how strongly attached ammonium is to biochar.

Using a different strategy, Wan et al. (2017) showed that phosphate-laden biochar, generated through sorption of phosphate in solution, improved lettuce seedling growth compared to un-laden biochar, suggesting that quantities of nutrients obtained purely through sorption may be adequate to promote plant growth when released back into soils. However, comparison with artificial fertiliser would be needed to interpret the results in the context of a potential replacement fertilization strategy. Interestingly, Li et al. (2016b) found that phosphate-laden biochar that had previously been magnetised and modified with MgO to improve sorption capacity also allowed slow release of phosphates in soil, which increased ryegrass growth compared to unamended soil and un-laden biochar in soil. The biochar was applied at a realistic rate (1% weight for weight), supporting the evidence that spent biochar could be used as a slow-release fertiliser soil amendment. Again, however, reliable comparison to artificial fertiliser is missing.

### 3.3 Nitrogen extraction by desorption from biochar

Direct application of nutrient-loaded biochar to soil is a promising area of research that should be explored further. However, it should be compared with the strategy of desorbing nutrients from magnetically-extracted biochar and using them separately in soil. Studies have shown that a range of methods can be used to achieve nitrogen desorption from biochar. Takaya et al. (2016a) used 0.01 M KCl to remove ammonium from biochar, but found only 5% could be removed after a 24-h incubation period. They attributed this result to ammonium being present in pores which were not well-accessed by KCl. However, Mia et al. (2017) used a different salt, CaCl_2_, at the same concentration (0.01 M) and achieved ~25% desorption on the first step, and after 5 desorption steps had achieved nearly 50% desorption. On the other hand, Wang et al. (2015d) had far greater success than both of these by using 2 M KCl, which was able to extract up to 99% of ammonium immediately when the pH was adjusted to ~3. Chintala et al. (2013), however, simply incubated biochar in deionized water that was adjusted to either pH 4 or pH 9 and achieved nitrate desorption of up to 90%. Overall, greater desorption occurred at pH 9, although there was a great deal of variability between different types of biochar, so optimum pH for desorption would depend on the biochar being used. Desorption is clearly possible, but how the desorbed ions can be used as a fertiliser has not yet been investigated. Magnetic biochar removed from agricultural soil is likely to have also sorbed a range of organic and inorganic compounds (even if it has been tailored to maximise ammonium sorption), and therefore the process of desorbing ammonium may also lead to a cocktail of components. For example, magnetic biochar has been shown experimentally to effectively sorb heavy metals such as arsenic (Wang et al., 2015a; Zhang et al., 2013), chromium (Han et al., 2016; Shang et al., 2016), cadmium (Wan et al., 2020; Yap et al., 2017) and lead (Rama Chandraiah, 2016). While this may not be problematic, there are potential risks associated with not analysing the solution content or processing it further to remove unwanted compounds. Firstly, is the issue of toxicity, as while pollutants such as heavy metals may not have been at toxic levels in the soil from which they were removed, there is the risk of concentrating them through the desorption process, and subsequently reapplying them at significantly greater concentrations. Furthermore, there is the issue of reduced efficiency, for example, by ammonium binding with other molecules in the desorption solution and not being available to plants when reapplied as fertiliser. Therefore, it is imperative that any process involving re-use of nitrogen that has been desorbed from magnetic biochar incorporates screening for levels of potentially toxic pollutants, and if necessary, further processing to remove unwanted interferences.

### 3.4 Magnetic biochar recycling

Research has shown that after desorption, magnetic biochar can be regenerated for use in successive rounds of sorption-desorption treatments. Wang et al. (2015b) desorbed 84.1% of Pb(II) from magnetic biochar using EDTA-2Na and found that for six subsequent rounds of sorption-desorption the sorption efficiency remained close to original levels. They also found an increase in surface area and pore volume, and no significant shifts in FTIR bands, suggesting that important physiochemical properties were not negatively affected. In addition, magnetic properties did not appear to be majorly affected. Similarly, sorption-desorption experiments of Cr (VI) by Shi et al. (2018) showed that by round six, 85% of the initial sorption capacity was retained. Using a similar methodology, Duan et al. (2017) concurred that efficiency remains high, showing that the adsorption capacity was unchanged after four rounds. There is, therefore, some evidence that biochar can be regenerated and reused with relatively unchanged properties. However, experiments for the specific pollutant, ammonium, are still required. Furthermore, studies have so far been performed in controlled aqueous environments. The effects may be different on magnetic biochar that has been retrieved from soils, where many other compounds could be present that may interact with the mechanisms involved in sorption/desorption. Also, biochar recycling has so far involved additional steps of filtering, washing, and drying regenerated biochar, which could add complexity and costs to the overall process. Further study should therefore consider realistic applications in the context of varying agricultural systems.

### 3.5 Magnetic separation of biochar from soil

**Fig. 4:**
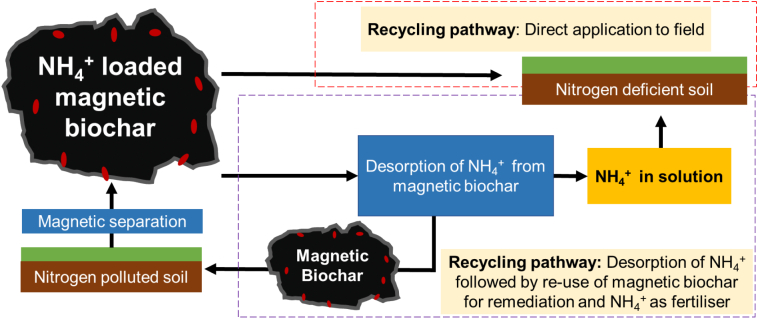
Two recycling pathways possible for reuse of magnetic biochar and ammonium after remediation of nitrogen polluted soil. ‘NH_4_^+^ loaded magnetic biochar’ refers to magnetic biochar that has been separated from soil after sorption of ammonium.

While biochar has been used as a soil pollutant remediation strategy via in-situ immobilisation, few studies have investigated the potential for magnetic biochar to sorb and remove pollutants from agricultural soils. The most common use of magnetic biochar has been for wastewater treatment where there is a demand for cheap and effective strategies to separate pollutants from water (Yan et al., 2014; Yap et al., 2017; Damdib et al., 2019). However, removing pollutant-loaded biochar from soil would come with similar advantages – it would prevent re-release of pollutants back into soil, the magnetic biochar could be recycled for further use and pollutants could be extracted for re-use in the agricultural system (for example, nitrates, ammonium, and phosphates could be recycled for fertiliser use) (Fig. 4).

For this to be possible, magnetic separation technology needs to be designed or adapted to suit extraction of magnetic biochar from agricultural soils. Studies have investigated small-scale recoverability of magnetic material from soil, with good levels of recoverability. Feng et al. (2016) showed that copper in sandy and organic soils was less bio-accessible when zero-valent iron (ZVI) was applied to soil and subsequently magnetically extracted with a rectangular hand magnet, compared to leaving ZVI in the soil. Furthermore, Li et al. (2020b) found that arsenic leaching from soil was significantly reduced using the same technique (although a u-shaped magnet was used for separation), but attributed this mainly to immobilisation of arsenic in soil as only 2% of arsenic was removed by magnetic separation. In contrast to this, Cui et al. (2019) were able to remove 75% of arsenic from sandy soil samples using a biomass-derived nanocomposite of As loaded onto small pieces of sponge, which were separated from soils with a small, flat circular magnet in laboratory experiments. The magnetic separation was more successful when the magnetic composite was loaded onto sponge, suggesting that magnetic biochar powder could be incorporated onto a cheap, porous material to improve subsequent removal from soil. However, the potential environmental hazards of adding any synthetic material additional to biochar must be carefully evaluated for this to become a viable strategy in soil remediation. Variability of soil properties is also likely to influence magnetic separation; for example, it would be expected that wetter soils may adhere to amendments more than drier soils. Little evidence exists to evaluate this hypothesis, although Feng et al. (2007) found that iron filings in water saturated soils had similarly high removal efficiency to iron filings in field capacity and air-dried soils (>90%) (with removal using a rectangular hand magnet), suggesting that magnetic amendments can be efficiently removed from a range of soil moisture levels. Other soil physical properties, such as the amount of clay in the soil, will also affect the ease at which magnetic biochar can be added and removed, therefore emphasising the need for magnetic biochar to be designed not only for specific sorption abilities but also for its application and removal in different soil types.

**Fig. 5:**
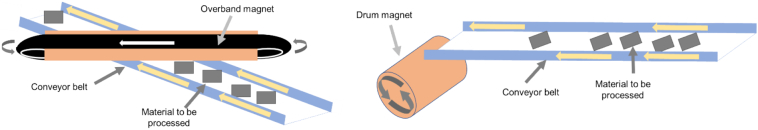
Typical set-ups of overband magnetic separators (left) and drum magnetic separators (right).

These studies give a promising indication that magnetic biochar extraction from soil could work as a pollutant remediation strategy, but scalability to field applications remains to be determined. To date, no known studies exist that have used MBES in field studies. Large-scale magnetic separation is, however, an established technology that is used widely in industries such as recycling, mining and mineral processing, power stations and ceramics (Bunting Magnetics, 2019). Effective separators include overband magnets, drum magnets and permanent magnetic roll separators (Fig. 5), where the typical process is a constant feed of material passed closely to a highly magnetic surface (which is itself being spun, rotated, or conveyed), allowing magnetic particles to be extracted (Svoboda and Fujita, 2003). However, the equipment is in a fixed location and the material to be processed is brought into the system externally; the challenge in agricultural soil application will be in developing in-field, moveable separation equipment that can process a continuous flow of soil with minimum environmental disturbance. Modification of existing equipment, such as tractor mounted rotary tillers, would be a potential low-cost method of integrating MBES into typical farm systems.

### 3.6 Summary

The current body of research is clearly an evolving and improving area, with good scope for a range of applications. However, there is a clear emphasis on water remediation. Arguably, there are added layers of complexity for soil remediation, such as environmental considerations relating to sensitive soil ecosystems, mechanical considerations relating to application and removal of magnetic biochar and biochemical considerations relating to interactions with the highly variable organic and inorganic components of soil. A major shortcoming in existing research is the short-term, small-scale nature of experiments, which although provide useful preliminary data for understanding the mechanisms of biochar in soil, fail to anticipate the added variability and complexity in real-world application. For soil remediation, the use of ‘designer’ magnetic biochar is a necessity due to the variability in sorption capacity for the range of pollutants that exist in soil and the differing impacts on soil chemistry and biology (Section 4). An example of how this could be used to assist both manufacturers and farmers has been demonstrated by an online decision support tool developed by the United States Department of Agriculture (USDA), the PNW Biochar Atlas (Phillips and Trippe, 2017). Soil properties and farm-specific goals are used by the software to generate a list of the best biochars types (for example, poultry litter biochar, 500 °C).

The majority of magnetic biochars produced in studies have shown good extractability from aqueous media using magnets, but studies using soil are limited, although some small-scale experiments provide promising evidence that it may be possible. A major challenge will be in developing equipment that will be able to remove magnetic biochar from soils at a large-scale. Fortunately, technology for magnetic separation is used effectively in industrial processes, such as waste processing, so mechanical and technical innovations could build on existing systems. Again, however, managing costs and scalability will be a crucial component of development.

## 4 Biochar interactions with soil ecosystems

### 4.1 Overview

Global biodiversity decline is widely-recognised as an issue in its own right, as well as being interlinked with other major concerns such as climate change and human health (IPBES, 2019). This trend is included in broader concerns surrounding deteriorating soil biodiversity, and as a result, the UK Government's 25 Year Environmental Plan contains a commitment to boost soil ecosystems (UK Government, 2018). Therefore, any actions that could potentially affect soil chemistry and biology need to be comprehensively assessed to mitigate against short and long-term negative impacts on the soil ecosystems. Biochar is one such example.

### 4.2 Potential effects on soil chemistry

While extensive research has been used to characterise magnetic biochar and to study its sorption capacity in aqueous settings, very little evidence exists to elucidate its interactions with soil components. However, an increasing volume of work has investigated un-modified biochar in soil, and while magnetic biochar will have different biochemical effects, general trends may still be relevant, and existing experimental methods could prove useful to develop research in this novel area. Understanding biochar-induced changes to soil properties is essential to determine its impact on wider soil parameters, such as nutrient cycling, biodiversity and plant health.

Soil pH is one variable that is affected by biochar. The presence of negatively charged functional groups is presumed to bind protons from soil, reducing the acidity of soil (Gul et al., 2015). Rees et al. (2014) determined there was an overall increase in soil pH (from 5.8 to 6.9) after 0.5 g of biochar (pH 9) was added to 4.5 g of soil (corresponding to redoxic cambisol) and 49.5 ml 0.01 M Ca(NO_3_)_2_ and shaken for a week, although a different soil, with lower concentrations of exchangeable metals, showed no significant change in pH. Obia et al. (2015) also found biochar (added at 1% w/w) increased the pH of acidic, sandy loam Acrisols, and the variability in alkalinity between different biochars caused very different levels of pH change (for example, soil pH increased by 0.2 units from one biochar type with a pH of 8.4, while soil pH increased by 2.3 units from another biochar with a pH of 9.8). Biochar has even been studied as a potential liming amendment (Hass et al., 2012) because of its effect on soil pH. Interestingly, in addition to pyrolysis temperature and feedstock, soil processes may act to enhance or limit the effect of biochar. For example, Dai et al. (2014) showed that biochar increased nitrification by microorganisms, which actually led to decreases in pH. Overall, therefore, magnetic biochar is likely to impact on soil pH, but the degree to which this occurs is highly dependent on its properties, as well as interactions with soil microorganisms.

Soil CEC has also been shown to be increased by biochar addition (Chen et al., 2011b; Jiang et al., 2012; Shi et al., 2017), due to enhanced availability of negatively charged functional groups. This is also related to the pH increase associated with biochar addition, as fewer protons are in competition with cations for binding to surface groups (Gul et al., 2015). The effect of higher CEC is likely to be a reduction in leaching of cations from soils, and therefore important nutrients for plants, such as Mg^2+^, Ca^2+^ and K^+^, are retained. Again, the main determinants for CEC are the physio-chemical properties of biochar, so altering the behavior of cations in soil may be possible through modification of biochar.

### 4.3 Biochar effects on soil microorganisms

Xu et al. (2014) found higher bacterial diversity in biochar-amended soils compared to control soils, which they attributed to the higher pH and C/N ratio. Nitrifying and denitrifying bacteria appeared to be stimulated by the biochar addition, with increased transcription of the nitrous oxide reductase gene (nosZ), leading to overall reductions in N_2_O emissions. Similarly, Liu et al. (2017) found an increase in three genera of phosphorus solubilising bacteria, with higher levels of phosphatase activity in biochar amended soil. Qian et al. (2019) concurred and reported increased abundance of a single P-solubilising species after biochar addition. Other studies have also found biochar to be beneficial to microorganisms (Jin, 1989; de Rozari et al., 2016; Mitchell et al., 2016; Solaiman et al., 2019). However, the proposed method of magnetically extracting N-loaded biochar from soil could reverse these effects. As soluble ammonium concentrations are reduced in the soil, nitrifying bacteria may undergo reductions in abundance, or, as soluble nitrate decreases, so could denitrifying bacteria. Future studies on magnetic biochar in soil should seek to include analyses of colonisation of magnetic biochar by nutrient-cycling microorganisms to elucidate these impacts. Furthermore, addition of biochar has also been shown to have negative effects on important soil microbes. For example, Warnock et al. (2010) found that arbuscular mycorrhizal fungal (AMF) abundance was decreased in some biochar treatments, primarily due to reductions in P availability, while Andrés et al. (2019) found overall reductions in microbial biomass and Dempster et al. (2012) found reductions in microbial activity.

Some direct interactions also occur between microorganisms and biochar. For example, hyphae of AMF have been shown to colonise biochar surfaces (Hammer et al., 2014; Vanek and Lehmann, 2015), possibly to gain access to nutrients. This, in turn, can act to transfer nutrients from sites that are inaccessible to plants to host plant roots. Magnetic extraction of biochar could offer new opportunities for experimental methods in this area, as biochar could be removed in a less invasive manner at desired time-points and subsequently screened for microbial colonisation. The interactions between magnetic particles and microorganisms must also be considered, as this could affect the stability and magnetism of magnetic biochar (for example, if magnetic particles are altered by microorganisms), or toxicity towards microorganisms could occur. Wu et al. (2021) found a significant reduction in microbial biomass after application of magnetic pig manure and eucalyptus residue biochar to soil compared to unmodified controls, possibly caused by iron oxides reducing phosphorus accessibility to microorganisms. On the other hand, microorganisms may benefit from nutrients such as iron that are provided by magnetic biochar, enhancing soil health. For example, Hori et al. (2015) isolated and identified particular groups of crystalline iron (III) oxide reducing bacteria from soil, finding novel iron (III) reducers in the *Geobacter* and *Pelobacter* genera, showing that certain microorganisms may benefit from the increased presence of magnetic particles. Interestingly, wider microbiological effects may result from increased success of iron (III) reducers in the presence of crystalline iron (III) oxides – for example, Qu et al. (2004) showed that methanogenesis was reduced in anoxic paddy soils, due to iron (III) reducers lowering the hydrogen partial pressure in soil to a level that could not be used by methanogens. However, this effect was far greater in iron (III) oxides of lower crystallinity, such as ferrihydrite and lepidocrocite than in more crystalline iron (III) oxides such as hematite. Given that more crystallised magnetic phases such as hematite, maghemite and magnetite are the more commonly occurring iron oxides found on magnetic biochar (Table 2), this effect may not be particularly prevalent; however, it demonstrates that additional soil processes must be considered where soil bacteria are affected by magnetic particles. Furthermore, this shows there are bacterial groups in soil that could cause physical and chemical changes to magnetic particles, but the resultant effect on biochar magnetism (if any) is yet to be investigated.

An additional issue relating to magnetic biochar addition may arise due to the formation of pollutants such as environmentally persistent free radicals (EPFRs) during magnetic biochar production. These can form on the surfaces of metals such as iron (Vejerano et al., 2011), a cation that is found in abundance on the surface of magnetic biochar. EPFRs may be subsequently released into the soil environment, posing risks to the soil ecosystems and human health. This issue was reviewed by Ruan et al. (2019), who acknowledged the potential benefits of EPFR production for contaminant degradation but stressed the necessity for clear assessments of the impacts on soil chemistry and biology. Magnetic biochar may therefore pose risks above and beyond those of unmodified biochar, and these must be assessed and weighed against its benefits.

Overall, the effects of biochar on soil microbes are strongly influenced by biochar properties. For example, a review by Gul et al. (2015) determined that manure or crop residue feedstocks promoted microbial abundance more than wood-derived feedstocks. The likely cause of this variation is differing effects on soil chemistry and availability of nutrients like N or P. Added complexity is introduced when the biochar is designed for sorption and removal of nutrients, so further research to elucidate these effects will be welcome in developing magnetic biochar for removal of pollutants from soil.

### 4.4 Biochar effects on soil fauna

Also key to soil health are animals, including arthropods, nematodes and annelids. Earthworms for example, are essential for cycling nutrients through soil through the mechanical effects of consumption and excretion, and interact closely with soil microorganisms (Bhadauria and Saxena, 2010). Studies have found mixed effects of biochar on earthworms. Malev et al. (2016) found that earthworms avoided biochar-amended soils above certain rates of application and were exposed to toxicity from polycyclic aromatic hydrocarbon (PAH) after 42 days of accumulation on biochar. On the other hand, Paz-Ferreiro et al. (2015) concluded that biochar did not affect earthworms, but found earthworms affected the microbial community, so indirect interactions may occur where biochar, earthworms and microorganisms are present in soil. Domene et al. (2014) tentatively suggested that biochar can enhance earthworm activity, but this is likely to be indirectly mediated by the increase in microorganisms. Oligochaetes, such as Enchytraeids, play a similar role in soils to earthworms, and in one study have been shown to be unaffected by biochar addition (Domene et al., 2015). The study did, however, find that an arthropod group, the Collembola, avoided biochar in soil regardless of the concentration. In contrast, Marks et al. (2014) determined that their wood biochar produced by both slow and fast pyrolysis stimulated collembolan reproduction, while their pine wood biochar produced by gasification could increase collembolan mortality. Again, microbial-mediated effects are hypothesised, but ascertaining the precise causes of stimulation/inhibition will be essential before biochar can be used as a sustainable amendment. Overall, the limited evidence suggests soil fauna to be less affected by biochar than soil microorganisms, but more research is needed to understand both short and long-term effects.

### 4.5 Biochar effects on agricultural plants

Plants are a major component of farming – food crops, cover crops, grassland, biofuel crops, trees, hedgerows, intercropping, wildflowers, and more all feature on farmland. As a soil amendment, biochar has been used predominantly to enhance crop growth, but its effect on the wider plant environment has not been explored. Three main mechanisms are likely to affect plants; alteration of soil chemistry, stimulation/inhibition of microorganisms and animals, and increasing/decreasing availability of essential nutrients. For example, dramatic pH changes could provide more/less favourable environments for plants to grow in, and this is likely to vary between plant species which have adapted to thrive in particular soil conditions. Also, introduction of metals such as iron could affect plant growth. For example, Wu et al. (2021) found that application of magnetic biochar significantly increased Fe plaque formation (by up to 75%) on *Phragmites australis* roots compared to unmodified biochar. Reduced plant growth was seen in these groups, but the authors concluded that inhibition of phosphorus uptake was more likely the cause of this than iron toxicity. Plants often have symbiotic relationships with bacteria and fungi, and if beneficial microorganisms such as AMF are stimulated by biochar, subsequent improvements in crop growth are likely to occur (Hammer et al., 2014; Shen et al., 2016). On the other hand, a reduction in beneficial microorganisms, or increases in detrimental microorganisms (e.g. soil-borne pathogens), could inhibit plant growth. However, changes to nutrient uptake are likely to have more immediate effects on plants after biochar addition. While increased availability of nutrients has been shown in experiments (Fox et al., 2014; Hammer et al., 2014; Vanek and Lehmann, 2015; Gao and DeLuca, 2018), some studies have shown the opposite effect (Warnock et al., 2010; Dempster et al., 2012). Furthermore, the application of biochar to remove nitrogen from soils risks causing nitrogen deficiencies if too much is removed. This could be countered by biochar application to field margins only, where the majority of nitrogen captured may be run-off from the cropped sections of the field (Fig. 1), although this could reduce nitrogen availability for nearby wildflowers, hedgerows, and trees. The aforementioned requirement for field-scale modelling and trials would therefore be relevant here in determining overall impacts on farm ecosystems.

### 4.6 Summary

The physio-chemical properties of biochar render it reactive with many components of the environment to which it is applied. This is clear in agricultural soils, with biochar causing minor to profound effects on microorganisms, animals and plants after both short- and long-term application. The complex interactions within soil ecosystems means these effects cannot be simply defined as ‘positive’ or ‘negative’ but must be studied at the field-scale to understand the impact of biochar on the soil biome. Furthermore, biochar can itself by affected by the soil community, which may consequentially affect its sorption dynamics over time, as demonstrated by Cui et al. (2018), who found fluctuations in antibiotic sorption during 60 days of biochar application to soil. This complexity is further compounded by the use of magnetic biochar and MBES, as an additional change to soil chemistry is likely to occur upon and after extraction. However, existing studies can be used to predict effects and develop methods for further soil-magnetic biochar research. For example, the identification of specific indicators relating to magnetic biochar impacts on soil health (such as iron toxicity or EPFR production) that can be used alongside an assessment protocol could allow biochar to be used in soils without causing environmental damage, following the method proposed by He et al. (2021).

## 5 Conclusions and future research

The field of biochar research is a continuously growing area, offering low-cost, innovative solutions to a range of problems, including climate change, pollution and soil nutrient deficiencies. Detailed characterisation of biochar, including FTIR, SEM, XRD, and BET analysis, has enabled researchers to establish that there exists a great variety of biochar physio-chemical properties, which in turn are strongly influenced by feedstock, pyrolysis conditions, and treatment. This has led to the idea that biochar can be tailor-made for specific applications to maximise efficiency, although more research is needed to fully understand the mechanisms involved. The use of biochar in pollutant sorption is one particular area where the ‘designer’ approach could be highly beneficial, as extensive study has shown that sorption capacity is strongly affected by biochar properties. Furthermore, novel treatments, like magnetisation, enhance the scope for opportunities in pollutant remediation. While magnetic biochar has shown promise as a strategy for water pollutant sorption and removal, its use for the same application in soil is an area of research that remains lacking. The potential benefits it could provide as a method to prevent nitrogen pollution, while increasing nitrogen-use-efficiency, means this novel subject area should be explored further. Furthermore, other pollutants of concern could be removed in a similar manner, so setting out robust experimental methods for magnetic biochar sorption in soil, and subsequent removal, will likely be of benefit to the wider field of pollutant remediation.

Considering certain gaps in the literature, some questions must be considered in order to develop future research.

a) Is microwave pyrolysis a more sustainable and cost-effective method of magnetic biochar production than conventional methods? Potential obstacles to large-scale biochar production on farms include the costs and logistics of pyrolysis. So far, the evidence suggests that microwave pyrolysis is highly-effective in terms of yield, biochar quality and energy efficiency. However, more research is needed to understand the effect of different microwave conditions on the biochar product, as well as how it can work alongside magnetisation.
b) What synthesis conditions are optimal to produce biochar for sorption of ammonium (e.g., feedstock, treatments, temperature)? Synthesis conditions are hugely influential on characteristics of biochar that are relevant to sorption, such as surface area and surface functional groups. However, different pollutants react differently with biochar and relatively little is known about trends relating to nitrogenous pollutants. Testing different conditions alongside biochar characterisation and sorption experiments will lead to an improved understanding of how to design biochar for N sorption.
c) What is the most effective method of magnetisation for the purpose of subsequent retrieval from soil? Different techniques of magnetisation have been used of varying complexity, but so far there does not appear to be any definite trends that indicate a superior method. Experiments that directly compare methods will be important in evaluating overall efficacy and cost-effectiveness. Furthermore, research should also focus on retrieval from soil media with experiments to understand factors such as the level of magnetism required, ideal particle size and potential loss of magnetic particles. Maximising retrievability of magnetic biochar will be just as important as maximising pollutant sorption capacity.
d) How does magnetic biochar affect sorption of ammonium compared to unmodified biochar? So far, magnetic biochar research has barely covered the area of nutrient sorption, and since magnetisation of biochar has been shown to affect sorption of other pollutants, such as metal ions, it is likely that ammonium sorption will also be affected. Firstly, therefore, experiments should be conducted to compare sorption by magnetic biochar compared to unmodified biochar, and secondly, these mechanisms should be investigated to allow improvement of magnetic biochar.
e) How are soil chemistry and biology affected by addition/removal of magnetic biochar? To maintain high environmental standards and farm profitability, any amendment to soils should not detriment the functioning of chemical and biological processes. As expected, due to the variability of both soils and biochar, research has found few consistent correlations between biochar addition and soil changes. This field itself therefore needs more attention. On top of this, the consequences of magnetic biochar addition and subsequent removal need to be understood, with particular focus on the changes brought about by alterations to nitrogen concentrations and availability in soils. This is a broad area that could include laboratory experiments, computer modelling and field trials. Additionally, potential value to biodiversity should be investigated, as demonstratable benefit to soil ecosystems could be a further metric for success.
f) Can spent magnetic biochar be recovered and re-used? Development of novel pollutant remediation methods should include research on scalability and sustainability as a major component. It is therefore essential that magnetic biochar can be used in a way where costs and environmental harm are minimised, and efficiency is maximised. This can be done through recycling of magnetic biochar, so experiments should examine reusability of magnetic biochar after removal from soils. In addition, where nitrogen has been removed by biochar sorption, its potential reuse should also be investigated.

## Funding

This work was supported by the 10.13039/501100000268Biotechnology and Biological Sciences Research Council Doctoral Training Partnership (Grant number BB/M008770/1).

## Declaration of competing interest

The authors declare that they have no known competing financial interests or personal relationships that could have appeared to influence the work reported in this paper.
